# Antimicrobial Prescribing Practices in Hospital Settings in Italy: A Retrospective Study

**DOI:** 10.3390/antibiotics12020218

**Published:** 2023-01-20

**Authors:** Francesco Napolitano, Concetta Paola Pelullo, Monica Lamberti, Giovanna Donnarumma, Gabriella Di Giuseppe

**Affiliations:** 1 Department of Experimental Medicine, University of Campania “Luigi Vanvitelli”, Via L. Armanni 5, 80138 Naples, Italy; 2 Department of Movement Sciences and Wellbeing, University of Naples “Parthenope”, Via Medina 40, 80133 Naples, Italy

**Keywords:** antibiotics, antimicrobial resistance, appropriateness, hospital, prescriptions, Italy

## Abstract

Background: This study aims to evaluate the antimicrobial prescribing practices in hospital settings in Italy, focusing on the appropriateness of antibiotic use. Methods: This study was carried out through a retrospective review of medical records of patients admitted in three public hospitals located in Campania Region (Italy) between 1 January and 31 December 2018. Results: More than one third (34.2%) of patients received at least one inappropriate antibiotic prescription (antibiotic administered and not indicated). Being female, having a >1 Charlson comorbidity index score, and having a longer hospital stay were significant determinants of an inappropriate antibiotic prescription. Instead, patients who had had a non-urgent hospital admission, an infection on hospital admission, and a microbiological culture test during hospital stay were significantly less likely to have an inappropriate prescription. When the antibiotic prescriptions were analyzed, in 26.6% of cases they were not indicated, while among the 687 antibiotic prescriptions with indication, incorrect choice of antibiotics (36.8%) was the most common reason of the inappropriateness. Conclusions: The findings of the study indicate that the inappropriate use of antibiotics continues to be a relevant issue in the hospital setting and specific interventions are needed to encourage a wider utilization of diagnostic tools to practice targeted therapies and to counter the antimicrobial resistance.

## 1. Introduction

Antimicrobial resistance (AMR) represents a main health issue worldwide and the World Health Organization (WHO) has defined it as one of the greatest threats to public health due to its epidemiological relevance and economic burden [1]. 

The growing increase of antibiotic-resistant bacteria, which impairs the effective treatment of related infectious diseases, results in a sequence of negative effects on patients and the community. Indeed, AMR determines an increase in the morbidity, duration, severity, and mortality of infectious diseases, as well as failure of surgical interventions with a consequent rise in healthcare costs due to the duration of hospitalization, frequent diagnostic investigations, and longer and complex antimicrobial treatments [2]. Furthermore, the effects of infectious diseases caused by multi-resistant microorganisms impacts on the overall well-being of the community due to the indirect costs for patients and their families, such as the loss of working days and disability [3]. 

To contrast AMR, the WHO has approved a global action on AMR to ensure the successful treatment of infectious diseases through several objectives, such as the improvement of understanding of AMR, the optimization of the use of antimicrobial agents, and the increase in the investments in the research on new antibiotics, diagnostic tools, and vaccines [4].

The latest Italian Antibiotic Use Report has shown that Italy is among the countries with the highest consumption of antibiotics and spread of multi-resistant microorganisms in Europe [5]. This concerning scenario has prompted the approval of a National Antimicrobial-Resistance Contrast Plan (PNCAR) [6], which indicates objectives and actions to be implemented at national, regional, and local levels in order to promote an effective contrast to AMR in Italy. 

It is well known that the non-optimal and inappropriate antibiotic use is one of the main causes of AMR and although most antibiotics are used for treating infections outside hospitals, in the period 2016–2020, hospital consumption of antibiotics showed an increase in Italy [7]. This is very worrisome, since the PNCAR has set the goal of a 5% reduction of antibiotic consumption in the hospital setting. Instead, there is room for an increase in the incidence of healthcare-associated infections (HAI) caused by resistant microorganisms.

Several investigations have been conducted to evaluate the prescriptions of antibiotics in the community and hospital settings [8,9,10] and to estimate the appropriateness of antibiotic prescription for infections caused by multidrug-resistant bacteria or for treatments of specific infectious diseases [11,12,13,14,15,16], while few experiences are available in Italy on antibiotic use for therapeutics reasons among hospital patients with or without infection on admission and the factors that can influence these antibiotic prescriptions [17,18]. The availability of data about the frequency and pattern of antimicrobial prescribing is crucial in order to achieve the success of action plans against AMR, to identify priority areas of intervention, and to implement training activities for healthcare workers to contrast the inappropriate antibiotic use and to provide useful information to improve antimicrobial prescriptions in the hospital setting.

Therefore, this study has two main objectives. The first is to evaluate the antimicrobial prescribing practices in hospital settings in Italy, focusing on the appropriateness of antibiotic use, and the second is to investigate which determinants could predict hospital inappropriate antibiotic use.

## 2. Results

Overall, 723 patients were enrolled in the study, and they had received 936 antibiotic prescriptions for therapeutic reasons. The main demographic and anamnestic characteristics of the study population are described in Table 1. More than half (52.4%) were males, the average age was 63.5 years (range: 34–89), the large majority (90.5%) had at least one chronic medical condition, and approximately two thirds (61.4%) had a >1 Charlson comorbidity index score. Moreover, 26.8% had low serum albumin, 19.2% an immunosuppression status, and 26.6% a clinical infection on admission. Urgent admission to hospitals involved one in five of the study participants (21.7%), 40.8% were hospitalized in general medicine wards, 21.6% in surgical specialties wards, the average length of hospital stay was 8.9 days (range: 3–30), 21.4% had had at least one hospital admission in the previous 12 months, and 3.6% had had an HAI during the hospital stay. 

Respiratory, urinary tracts, and skin and soft tissue were the most common sites of infection, resulting in 40.3%, 21.5%, and 13.1% of all infections, respectively. A microbiological culture test was performed only in 94 patients (13%) and the more frequently isolated microorganisms were *Escherichia coli* (23.4%), *Staphylococcus aureus* (20.2%), and *Klebsiella pneumoniae* (11.7%).

Out of all 723 patients who received antibiotics for therapeutic reasons, 510 had received one and 213 more than one antibiotic prescription for a total of 936 antibiotic prescriptions recorded. The groups of antibiotics more frequently prescribed were third generation cephalosporins (26.7%), penicillin (22.5%), fluoroquinolones (17.5%), and carbapenems (10.3%). Overall, more than one third of patients (34.2%) received at least one inappropriate antibiotic prescription (antibiotic administered and not indicated), and 26.7% and 15% of them received two and three inappropriate prescriptions, respectively. 

The results of univariate analysis showed that a significantly higher frequency of inappropriate antibiotic prescriptions for therapeutic reasons was observed in older patients (t = −3.96, *p* < 0.001), in those who had had an urgent hospital admission (68.1% vs. 24.7%; χ^2^ = 23.1, *p* < 0.001), with longer hospital stay (t = −2.91, *p* < 0.003), and without an infection on hospital admission (42% vs. 11.8%; χ^2^ = 56.3, *p* < 0.001), whereas no difference was found for the different wards of hospital stay. Moreover, the following anamnestic characteristics of patients were also associated with receiving inappropriate antibiotic prescription: not having an immunosuppression status (33.6% vs. 23.7%; χ^2^ = 8.31, *p* = 0.004), having a chronic medical condition (χ^2^ = 22.3, *p* < 0.001), having a higher Charlson comorbidity index score (39.2% vs. 26.2%; χ^2^ = 12.9, *p* < 0.001), and not having a microbiological culture test during hospital stay (36.7% vs. 17%; χ^2^ = 14.2, *p* < 0.001) (Table 1). 

The results of stepwise logistic regression model performed to estimate predictors of inappropriate antibiotics prescribing are shown in Table 2. Being female (OR = 5.26; 95% CI 2.95–9.39), having a >1 Charlson comorbidity index score (OR = 2.36; 95% CI 1.28–4.35), and having a longer hospital stay (OR = 1.24; 95% CI 1.16–1.33) were significant independent determinants of an inappropriate antibiotic prescription. Instead, patients who had had a non-urgent hospital admission (OR = 0.11; 95% CI 0.05–0.21), an infection on hospital admission (OR = 0.02; 95% CI 0.01–0.06), and a microbiological culture test during hospital stay (OR = 0.36; 95% CI 0.18–0.73) were significantly less likely to have an inappropriate antibiotic prescription.

Table 3 shows the reasons of the inappropriate antibiotic prescriptions according to indication, drug choice, dose, duration, and route of administration. No indication for the antibiotic prescription accounted about a quarter of the reasons of the inappropriateness (26.6%), while among the 687 antibiotic prescriptions with indication, incorrect choice of antibiotics (34.3%) was the most common reason of the inappropriateness, followed by incorrect doses (18.6%) and excessive length of the antibiotic therapy (16.5%). Among the 236 inappropriate prescriptions due to an incorrect choice of antibiotic, the class of antibiotics more frequently used inappropriately were third generation cephalosporins (36.8%), fluoroquinolones (30.1%), and aminopenicillins (20.3%). 

Among the selected patients, 226 (31.2%) underwent a surgical procedure and 190 (87.6%) of them had received antibiotic prescriptions for prophylaxis purpose. All 178 patients eligible for the prophylaxis received antibiotics, whereas among the 48 procedures for which the surgical antibiotic prophylaxis (SAP) was not indicated, more than half (52.1%) received antibiotics. Overall, only 56 SAP (24.8%) were appropriate in accordance with indication, choice of the antibiotic, timing, and doses recommended by the guidelines. The antibiotics most frequently used inappropriately were ceftriaxone (17.9%) and ceftazidime (12.1%).

## 3. Discussion

This study provides relevant information on hospital antibiotic prescribing practices in Italy, where the levels of antibiotic utilization and spread of multi-resistant microorganisms are higher compared to other European countries. The results could be useful for the development of specific measures to contrast inappropriate antibiotic use and AMR in the hospital setting.

This investigation has three key findings. A first key finding was that approximately one third of patients (34.2%) had received inappropriate antibiotic therapies since there was no indication. The comparison of this rate of inappropriateness with previous studies conducted in Italy and in other countries is difficult due to the different study criteria, healthcare setting, and characteristics of the study population. Despite these differences, similar results were found in two previous investigations where 33% of antibiotic prescriptions were not appropriate in a Swiss tertiary care hospital [19] and 32.7% of prescriptions were evaluated as inappropriate in emergency departments in Australia [20]. Lower rates of inappropriate prescriptions have been reported in a Dutch university hospital (29.3%) [21], whereas a higher frequency, ranging from 53.8% to 79.8%, were found in medical, surgical, and intensive care units in a previous investigation conducted in the same geographical area [22]. These data suggest that there is room for the improvement of adherence to antibiotic prescribing guidelines through the implementation of effective interventions. It is well known that antimicrobial stewardship programs (ASP) have a positive impact on the antibiotic use, and it could improve the hospitalization outcomes such as reduction of infectious diseases due to multidrug-resistant microorganisms, lengths of stay, readmission rate, and patients’ disability and mortality [23,24,25,26]. Therefore, it is essential that healthcare services promote ASP in all healthcare facilities and settings, since several investigations demonstrated that the participation in ASP improves the appropriateness of antibiotic prescriptions [27,28,29]. 

A second key finding was that, when the single antibiotic prescriptions were analyzed, no indication for antibiotic use accounted for about a quarter of the reasons of the inappropriateness. Indeed, 26.6% of the antibiotics used inappropriately were prescribed to patients who did not have any evidence of infectious disease in the medical record. A possible reason is that physicians at the admission or during the hospitalization start antibiotic treatment even if they are unsure of the diagnosis and, therefore, they do not modify the treatment. This is a very unsafe practice because the unnecessary use of antibiotics may generate AMR, while this behavior should be limited given that Italy is one of the European countries where the consumption of antibiotics is higher, and inappropriate practices regarding antibiotic use has been observed both in hospital and in other healthcare settings [11,13,30,31]. 

The latest Italian Antibiotic Use Report has shown that inappropriate use of antibiotics was between 25% and 30% and the main reasons of inappropriateness were the treatment in the adult population of upper respiratory tract infections and uncomplicated lower urinary tract infections [7]. Moreover, other reasons for inappropriate antibiotic use in Italy have been found to be unnecessary antimicrobial prescription in hospital [30] and antibiotic use without the prescription of a physician [31,32,33].

A third key finding was that the results of multivariate logistic regression analysis showed that patients who had an infection on hospital admission and a microbiological culture test during the hospital stay were significantly less likely to receive an inappropriate antibiotic prescription. This result can be explained by the fact that physicians in our sample prescribe antibiotics when they suspect an infection, and they confirmed the infectious diseases and isolates with the microbiological testing. This finding underlines the usefulness of microbiological tests to implement a correct use of antibiotics, even if only 13% of patients with infections had had a test during the hospital stay. In the global action plan on antimicrobial resistance adopted by WHO [4], a key strategy was the optimization of the antimicrobial use and the increase in the investment on research on rapid diagnostic tools to contrast the AMR. Therefore, the microbiological tests are essential in the management of antibiotic therapies in healthcare facilities. In addition, the routine application of antibiograms is of help in identifying the levels of sensitivity and resistance to antibiotics of the microorganisms and for the choice of appropriate antibiotic therapy by physicians. In this study, the finding that only a small proportion of patients had received a microbiological test underscores the need to investigate the reasons for the poor use of diagnostic microbiological tools in order to provide useful information to implement more effective training for physicians to improve the appropriate use of testing and antimicrobials. Moreover, although evaluation of the SAP was not a priority objective of this study, the results showed that a large majority of SAP was inappropriate according to the Italian guidelines. Therefore, there is a need for health managers and healthcare professionals to be train in more careful antibiotic use according to the available guidelines in order to improve appropriate SAP. 

In conclusion, the results of the present study indicate that the inappropriate use of antibiotics continues to be a relevant issue in the hospital setting. Therefore, it is imperative to implement the adherence of physicians to the guidelines on antibiotic use and the participation in ASP in order to improve the appropriateness in antimicrobial prescription and to reduce the use of broad-spectrum antibiotics. Moreover, specific interventions are needed to encourage a wider utilization of diagnostic tools in order to practice targeted therapies and to counter the AMR.

### Limitations

For a correct interpretation of the results, some limitations of the study should be considered. First, due to the retrospective study design, data were only retrieved by medical records. Indeed, it is possible that some information, such as medication prescriptions, reasons for the antibiotic use, and information on the patients’ risk profiles, has not been accurately reported in the medical records by the healthcare professionals. Therefore, it cannot be excluded that the rate of appropriateness of antibiotic use may be underestimated due to the lack of information in the medical records and consultation with a physician that did not allow us to clarify the reason of several antibiotic prescriptions with no indication. Second, data were collected only in three hospitals in the Campania region. The characteristics of the hospitals may not be representative of all hospitals in Italy, and this may limit the generalizability of the study findings. Despite these limits, this investigation has several strengths, such as a large sample size and careful data collection for a one-year period in the selected hospitals. Therefore, we are confident that the results of this study are valid and provide important information on antimicrobial prescribing practices for inpatients.

## 4. Materials and Methods

### 4.1. Setting, Study Design, and Sampling

This study was carried out through a retrospective review of medical records of patients admitted in three public hospitals located in Campania Region (Italy) between 1 January and 31 December 2018. The minimum sample size consisted of 480 subjects, and it was calculated assuming a 30% prevalence of patients who have received inappropriate administration of antibiotics, a 95% confidence interval, and a 5% error. In order to increase the precision of the results, a larger sample of patients was recruited. 

### 4.2. Study Procedure, Recruitment, and Instrument for Data Collection

Before starting the study, the Heads of the selected hospitals were contacted through a letter to obtain the approval to conduct the study and to describe the objectives of the investigation and the methods of collecting the information. After access to medical records was granted, the research team received the medical records in a secure electronic format from the medical records managers of the selected hospitals. Then, all information was reviewed and summarized on a standardized case report form by two authorized investigators not directly involved in patients care who consulted the medical records assuring the anonymity and confidentiality of the collected data. The medical records of patients fulfilling the following inclusion criteria were retrieved: (a) aged 18 years or above; (b) admitted for at least one day in medical or surgical wards; and (c) having received at least one antibiotic prescription for therapeutic reasons during their hospital stay. 

The following data were collected for each inpatient: age, gender, weight, height, date and diagnosis of hospital admission, ward and length of the hospital stay, hospitalization in the previous 12 months, smoking status, Charlson comorbidity index, type and number of chronic conditions, immunosuppression and nutrition status, previous allergies to antibiotics, clinical infection(s) on admission, microbiological culture test and antibiogram performed during the hospital stay, details about the antibiotic prescriptions (indication, type, timing, length, dose and route of administration of antibiotic therapy), and presence of HAI according to the criteria of the European Centers for Disease Control and Prevention [34]. Moreover, the following information were also collected for surgical patients: type of surgical procedure, surgical wound classification, ASA score, type of anesthesia, undergoing endoscopic surgery, implant of prosthesis, length of surgery, and details of SAP (type, timing, duration, dose, and route of administration).

Appropriateness of antibiotic therapies was assessed according to the Guidelines for the implementation of Antimicrobial Stewardship programs and for the local implementation of antibiotic therapy protocols of the Campania region, and the national and international guidelines on the antibiotic treatments and preoperative prophylaxis [35,36,37,38]. The antibiotic prescriptions for therapeutic reasons and the SAP were evaluated as appropriate if the indication, choice of the antibiotic, the timing, the dose(s), and the length of administration were in accordance with the guidelines.

### 4.3. Pilot Study and Ethical Statement 

The data collection instrument was pretested on a random sample of 25 medical records not included in the final sample, and the necessary changes were made before starting the study.

The study protocol was approved by the Ethical Committee of the Teaching Hospital of the University of Campania “Luigi Vanvitelli” (approval number 215/2019).

### 4.4. Statistical Analysis

Statistical analyses were performed using Stata version 15 software [39] and were conducted in two stages. First, bivariate analysis was carried-out to evaluate the effect of the independent variables on the inappropriate antibiotic therapy in hospital patients using chi-square and Student’s *t*-test for categorical and continuous variables, respectively. Subsequently, a multivariate stepwise logistic regression analysis was performed, including in the models the variables with a *p*-value ≤ 0.25 at the bivariate analysis according to Hosmer and Lemeshow’s model building strategy [40], to investigate the independent characteristics associated with inappropriate antibiotic prescriptions for therapeutic reasons during the hospitalization (no = 0; yes = 1). In the stepwise logistic regression model the following independent variables were included: age (continuous), gender (male = 0; female = 1), type of hospital admission (urgent = 0; non-urgent = 1), ward of hospital stay (general medicine = 1; medical specialties = 2; general surgery = 3; surgical specialties = 4), length of hospital stay (continuous, in days), having a clinical infection on hospital admission (no = 0; yes = 1), immunosuppression status (chemotherapy and long term steroids use) (no = 0, yes = 1), low serum albumin (<3.5 g per deciliter) (no = 0, yes = 1), number of chronic medical conditions (0 = 0; 1 = 1; >1 = 2), Charlson comorbidity index score (0–1 = 0; >1 = 1), microbiological culture test (no = 0; yes = 1). The significance levels for the exclusion and inclusion of variables in the model were 0.4 and 0.2, respectively. All inferential tests were performed through the execution of a bilateral hypothesis test with the statistical significance level of *p* values equal to or less than 0.05. The results of multivariate regression analyses were reported as odds ratios (ORs) and 95% confidence intervals (CIs). 

## Figures and Tables

**Table 1:** Demographic and anamnestic characteristics of the study population.

Characteristic	Total *n* = 723		Patients Receiving at Least One Inappropriate Antibiotic Prescription *n* = 247	
	***n***	**%**	***n***	**%**
Gender				
Female	344	47.6	111	32.2
Male	379	52.4	136	35.9
			χ^2^ = 1.36; *p* = 0.225	
Age, years	63.5 ± 13.5 (34–89) *		66.2 ± 11.3 (42–87) *	
			t = −3.96; *p* < 0.001	
Type of admission				
Non-urgent	566	78.3	140	24.7
Urgent	157	21.7	107	68.1
			χ^2^ = 23.1; *p* < 0.001	
Ward of hospital stay				
General medicine	295	40.8	95	32.2
Medical specialties	130	18	45	34.6
General surgery	142	19.6	51	35.9
Surgical specialties	156	21.6	56	35.8
			χ^2^ = 0.92; *p* = 0.821	
Length of hospital stay, days	8.9 ± 4.1 (3–30) *		9.6 ± 2.9 (5–17) *	
			*t*-test = −2.91; *p* = 0.003	
Having a clinical infection on admission				
No	536	63.4	225	42
Yes	187	26.6	22	11.8
			χ^2^ = 56.3; *p* < 0.001	
Immunosuppression status				
No	584	80.8	214	33.6
Yes	139	19.2	33	23.7
			χ^2^ = 8.31; *p* = 0.004	
Low serum albumin				
No	570	73.2	201	35.2
Yes	153	26.8	46	30.1
			χ^2^ = 0.87; *p* = 0.261	
Number of chronic medical condition				
0	69	9.5	8	11.5
1	259	35.8	108	41.7
>1	395	54.7	131	33.2
			χ^2^ = 22.3; *p* < 0.001	
Charlson comorbidity index score				
0–1	279	38.6	73	26.2
>1	444	61.4	174	39.2
			χ^2^ = 12.9; *p* < 0.001	
Hospital admission in the previous 12 months				
No	568	78.6	196	34.5
Yes	155	21.4	51	32.9
			χ^2^ = 1.12; *p* = 0.326	
Having a healthcare-associated infection (HAI)				
No	697	96.4	239	34.3
Yes	26	3.6	8	30.8
			χ^2^ = 2.32; *p =* 0.71	
Infection site ^				
Respiratory	191	40.3	43	22.5
Urinary	102	21.5	21	20.6
Skin and soft tissue	62	13.1	12	19.4
Gastrointestinal	33	6.9	5	15.1
Cardiovascular	23	4.8	3	13
Bloodstream	19	4.1	2	10.5
Other	44	9.3	4	9.1
			χ^2^ = 8.34; *p =* 0.264	
Microbiological culture test				
No	629	87	231	36.7
Yes	94	13	16	17
			χ^2^ = 14.2; *p* < 0.001	

* Mean ± Standard deviation (range). ^ Among those with information on infection site in medical records (*n* = 474).

**Table 2:** Results of multivariate logistic regression analysis to investigate the factors associated with the inappropriate antibiotic prescriptions in hospital patients.

Variable				
Model. Patients Receiving at Least One Inappropriate Antibiotic Prescription (Sample Size = 723)				
Log Likelihood = −308.92, χ^2^ = 310.66(8 df), *p* < 0.0001	OR	SE	95% CI	*p*
Gender				
Male	1 *			
Female	5.26	1.55	2.95–9.39	<0.001
Type of hospital admission				
Urgent	1 *			
Non urgent	0.11	0.03	0.05–0.21	<0.001
Clinical infection on hospital admission				
No	1 *			
Yes	0.02	0.01	0.01–0.06	<0.001
Length of hospital stay (continuous)	1.24	0.04	1.16–1.33	<0.001
Microbiological culture test				
No	1 *			
Yes	0.36	0.13	0.18–0.73	0.005
Charlson comorbidity index score				
0–1	1 *			
>1	2.36	0.74	1.28–4.35	0.006
Number of chronic medical condition				
0	1 *			
1	1.91	0.77	0.87–4.19	0.107
>1	Backward elimination			
Immunosuppression status				
No	1 *			
Yes	1.89	0.75	0.87–4.11	0.110
Age (continuous)	Backward elimination			
Ward of hospital stay	Backward elimination			
Low serum albumin	Backward elimination			

* Reference category.

**Table 3:** Reasons for inappropriate antibiotic prescription according to indication, drug choice, dose, duration, and route of administration.

Antibiotic Prescriptions for Therapeutics Reasons	Total *n =* 936	
	*n*	%
No indication (inappropriate)	249	26.6
Inappropriate drug choice *	236	34.3
Inappropriate dose *	128	18.6
Inappropriate duration *	113	16.5
Inappropriate route of administration *	28	4.1

* Among the 687 antibiotic prescriptions with indication.

## Data Availability

The data presented in this study are available on request from the corresponding author.
